# Pathological Hyperinsulinemia and Hyperglycemia in the Impaired Glucose Tolerance Stage Mediate Endothelial Dysfunction Through miR-21, PTEN/AKT/eNOS, and MARK/ET-1 Pathways

**DOI:** 10.3389/fendo.2021.644159

**Published:** 2021-04-23

**Authors:** Ran Liu, Shilin Guan, Zhongai Gao, Jingyu Wang, Jie Xu, Zhaohu Hao, Yi Zhang, Shaohua Yang, Zhenhong Guo, Juhong Yang, Hailin Shao, Baocheng Chang

**Affiliations:** ^1^ NHC Key Laboratory of Hormones and Development, Tianjin Key Laboratory of Metabolic Disease, Chu Hsien-I Memorial Hospital & Tianjin Institute of Endocrinology, Tianjin Medical University, Tianjin, China; ^2^ Tianjin Fourth Central Hospital, The Fourth Central Hospital Affiliated to Nankai University, The Fourth Central Clinical College, Tianjin Medical University, Tianjin, China

**Keywords:** endothelial dysfunction, hyperinsulinemia, hyperglycemia, impaired glucose tolerance, insulin resistance, high glucose concentrations, miR-21

## Abstract

**Background:**

Impaired glucose tolerance (IGT) is an important prediabetic stage characterized by elevated concentrations of glucose and insulin in the blood. The pathological hyperglycemia and hyperinsulinemia in IGT may regulate the expression of microRNA-21 (miR-21) and affect the downstream insulin signaling pathways, leading to endothelial cell dysfunction and early renal damage.

**Methods:**

The individual and combined effects of insulin and glucose were investigated using human glomerular endothelial cells (HGECs). The expression levels of miR-21, and PTEN/AKT/eNOS and MAPK/ET-1 pathway proteins in the treated cells were measured. The levels of nitric oxide (NO) and endothelin-1 (ET-1) secreted by the cells were also measured. The role of miR-21 in mediating the regulatory effects of insulin and glucose was assessed by overexpression/inhibition of this miRNA using mimics/inhibitor.

**Results:**

High (>16.7 mmol/L) concentration of glucose upregulated the expression of miR-21, leading to the activation and inhibition of the PTEN/AKT/eNOS and MAPK/ET-1 pathways, and upregulation of NO and downregulation of ET-1 secretion, respectively. High (>25 ng/mL) concentration of insulin downregulated the expression of miR-21, and lead to the activation of the MAPK/ET-1 and inhibition of the PTEN/AKT/eNOS pathway, thereby upregulating the expression of ET-1 and downregulating the secretion of NO. MiR-21 was observed to play a key role by directly controlling the activation of the insulin signaling pathways when the cells were cotreated with different concentrations of insulin and glucose. The expression of miR-21 was found to be dependent on the relative concentration of insulin and glucose. Under simulated conditions of the IGT stage (8.3 mmol/L glucose + 50 ng/mL insulin), the inhibitory effect of high insulin concentration on miR-21 expression in the cells attenuated the activation by high glucose concentration, resulting in the downregulation of miR-21, upregulation of ET-1 and downregulation of NO secretion.

**Conclusion:**

Taken together, these results indicate that high insulin and glucose concentrations regulate the secretory function of glomerular endothelial cells in opposite ways by regulating the expression of miRNA-21. Pathological concentrations of insulin and glucose in the IGT stage may lead to a decrease in miR-21 expression, thereby disordering the secretion of vasoactive factors, resulting in renal tubule ischemia.

## Introduction

Diabetic kidney disease (DKD) has become a leading cause of end-stage renal disease (ESRD) with the increase in morbidity associated with diabetes (1). The early prevention and treatment for DKD is difficult owing to the lack of means for sensitive and specific diagnosis. Microalbuminuria (MAU) is the main clinical criterion for the detection of DKD. However, only a few patients with MAU can recover to have normal MAU levels or maintain the current level after treatment, and 30%–40% of patients gradually develop macroalbuminuria, and even ESRD (2). The main reason for this could be the occurrence of pathophysiological changes associated with DKD (including glomerular basement membrane thickening, podocyte damage, and mesangial expansion) in the kidney even before the onset of MAU (3). The cause of this early renal damage and the mechanism responsible for it remain unclear. Therefore, investigations on the early pathophysiological changes in DKD are of great importance for its early prevention and treatment.

The development of type 2 diabetes mellitus (T2DM) is a gradual process starting from normal glucose tolerance (NGT), through impaired glucose tolerance (IGT), to hyperglycemia. The IGT stage represents an intermediate stage during which plasma glucose levels are above normal but do not meet the criteria for diabetes. As an important stage in the development of prediabetes, IGT can also be an independent risk factor for kidney diseases (4). It has been found that MAU already exists in 13.1% of patients with IGT (5), indicating that IGT might be independently associated with the pathology of DKD. In a previous study, we found that the indicators of urinary tubule injury were significantly increased in patients with IGT, and estimated glomerular filtration rate (EGFR) and glomerular hyperfiltration were also obviously increased in these patients (6). Therefore, it is important to further study the alteration of kidney structure and function during the IGT stage. In our previous research, the pathological mechanism of IGT nephropathy were studied in OLETF rat, which manifested by obesity, insulin resistance, spontaneous hyperglycemia, and proteinuria, and is an ideal animal model of DKD in human. OLETF rats were given a high-fat diet at 4 weeks age. At 32 weeks age, the OLEFT rats had developed pathological characteristics of IGT(peak level of blood glucose > 16.8 mmol/L or 120 min blood glucose level > 11.1mmol/L, and hyperinsulinemia). The main presentation of renal injury in the IGT stage in OLETF rats is tubular injury, which includes the irregular array of tubular epithelial cells, the shedding off of brush-border tubular epithelial cells, and infiltration of inflammatory cells (7). Therefore, we propose the renal tubular damage, which occurs in the IGT stage, as “IGT nephropathy”. However, the mechanism of IGT nephropathy is still unclear. The core pathophysiological changes in the IGT stage are insulin resistance and compensatory hyperinsulinemia (8). Although the compensatory elevation in insulin concentration helps control blood glucose levels, it may be harmful to the heart, kidneys, and other tissues (9, 10). Endothelial cells are an important part of renal microcirculation. Changes in metabolism (such as hyperglycemia), immunity, and hemodynamics can directly lead to the damage of the vascular endothelium (11, 12). The injury and dysfunction of endothelial cells is an initial step in DKD (13). Studies have shown that insulin may affect the release of vasoactive substances by endothelial cells through the PI3K/Akt/eNOS and RAS/MAPK/ET-1 pathways, and then affects the blood flow through the surrounding capillaries (14–16).

Therefore, we speculate that the pathogenic factors of the IGT stage (hyperinsulinemia and hyperglycemia) can affect the function of capillary endothelial cells through the PI3K/Akt/eNOS and MAPK/ET-1 pathways, and participate in the progress of renal tubular ischemia and hypoxia, leading to IGT nephropathy.

MicroRNAs are endogenously produced short noncoding RNAs (17). MiR-21 plays an important role in the pathological process of diabetic nephropathy. The expression of miR-21 in the renal tissue was found to change in the early stage of diabetes, and it was shown to be involved in the development of DKD (18–20). However, whether miR-21 is involved in IGT nephropathy remains to be studied.

In this study, to investigate the role of miR-21 in IGT nephropathy and to explore the possible mechanism, we simulated the characteristics of blood glucose and insulin in the IGT stage *in vitro* using the glomerular endothelial cells and observed the changes in the expression of miR-21 and proteins in the downstream pathways and endothelial cell function.

## Materials and Methods

### Cell Culture and Intervention

Primary human glomerular endothelial cells (HGECs, ScienCell Company, Beijing Yuhengfeng Agency, China) were cultured in Endothelial Cell Medium containing 10% fetal bovine serum (Endothelial Cell Medium and serum were from ScienCell Company, Beijing Yuhengfeng Agency, China). The cells were used within six passages. Cells in the high insulin concentration group were treated with medium containing different concentrations of insulin: 7.14 ul, 14.28ul, 35.7ul, 71.4ul of Novolin insulin solution (Novo Nordisk, Copenhagen, Denmark) was added to 2 ml ECM medium separately(The ECM medium did not contain any insulin) to form 5 ng/ml, 10ng/ml, 25ng/ml, 50ng/ml high concentration insulin medium. Cells in the high glucose concentration group were treated with medium containing anhydrous glucose (Solarbio, Beijing, China) at 8.3, 11.1, 16.7, or 33.3 mmol/L. D-Mannitol (Solarbio, Beijing, China) was used as a control for osmolarity. HGECs were transfected with miR-21 mimics or miR-21 inhibitor (GenePharma, Shanghai, China) using the Lipofectamine 2000 reagent (Invitrogen, Carlsbad, CA, USA). The transfection was performed under strictly aseptic conditions according to the manufacturer’s instructions. The effectiveness of miR-21 mimics and inhibitor was verified by PCR after transfection (**Figures 4A, E**).

### Quantitative Real-time Polymerase Chain Reaction

Total RNA was extracted from HGECs using miRNeasy Mini Kit (Qiagen, Hilden, Germany). The first-strand cDNA was constructed using a Taqman MicroRNA Reverse Transcription Kit (ABI, Carlsbad, CA, USA). The primers used in this study were obtained from Genscript Corp (Sangon, Shanghai, China), and their sequences are listed in **Table 1**. U6 was used as an endogenous control for the miRNAs. The relative gene expression was calculated using the ΔΔCt method. (The Ct value of U6 in each group is stable as listed in the **Supplemental Materials**).

**Table 1:** Primers sequences used in qRT-PCR.

Gene^*^	Primer	
has-miR-21-5p stemloop	5’-GTCGTATCCAGTGCAGGGTCCGAGGTATTCGCACTGGA TACGACCTACGC-3’	
hsa miR-21-5p	Forward	5’-GGTCCTTATTGCTTAAGAATACGCG-3’
	Reverse	5’-CCAGTGCAGGGTCCGAGGT-3’
hsa U6	Forward	5’-TGCGGGTGCTCGCTTCGGCAGC-3’
	Reverse	5’-CCAGTGCAGGGTCCGAGGT-3’

*hsa, human.

### Western Blot Analysis

The cells were lysed in rapid cell lysis buffer (Solarbio, Beijing, China). The supernatant obtained after centrifugation of the cell homogenate was collected. Samples were resolved by SDS-PAGE and the proteins were transferred onto PVDF membranes (Thermo, CA, USA). The membrane was blocked with nonfat milk and incubated overnight at 4°C with the primary antibodies against phosphatase and tensin homologue on chromosometen (PTEN; diluted to 1:1000, Cell Signaling), AKT (diluted to 1:1000, Cell Signaling), phospho-AKT (P-AKT; diluted to 1:1000, Cell Signaling), p38 mitogen-activated protein kinase (P38-MAPK; diluted to 1:1000, Abclonal), phospho-p38 mitogen-activated protein kinase (P-P38 MAPK; diluted to 1:1000, Abclonal), endothelial nitric oxide synthase (eNOS; diluted to 1:1000, Abcam), phospho-endothelial nitric oxide synthase (P-eNOS; diluted to 1:1000, Abcam), and endothelin-1 (ET-1; diluted to 1:500, Abcam). Thereafter, the membranes were washed with TBST buffer and incubated with the respective mouse or rabbit peroxidase-conjugated secondary antibody (Sungene Biotech, Tianjin, China). The protein bands were visualized using electrochemilminescence (ECL) blotting detection reagent (Advansta, Menlo Park, CA, USA) and quantified by densitometry using the Image J software.

### Enzyme-Linked Immunosorbent Assay

HGECs were seeded in a 24-well culture plate (BIOFIL, Guangzhou, China) at a density of 1 × 10^5^ cells per well and treated as described above. The cell medium was aspirated and centrifuged. The concentration of ET-1 was detected using ET-1 enzyme-linked immunosorbent assay (ELISA) kit (Fermentas, USA) following the manufacturer’s protocol. The absorbance was determined at 450mm as the reference wavelength by a multiscan Spectrum spectrophotometer (Synergy LX, Biotek, Germany); A ET-1 reference curve was established for each assay and ET-1 concentrations were calculated using the reference curve.

### Nitric Oxide Assay

HGECs were seeded in a 96-well culture plate (BIOFIL, Guangzhou, China) and treated as described above. The cell medium was collected and centrifuged. The concentration of NO was detected using Nitric oxide Assay Kit (Jiancheng Company, Nanjing, China) following the manufacturer’s protocol. The absorbance was determined at 550mm as the reference wavelength by multiscan Spectrum and the NO concentration was calculated according to the following formula:

$$\text{NO concentration }(\mu \text{mol}/\text{L})=\frac{\text{test group OD}-\text{blank group OD}}{\text{standard group OD}-\text{blank group OD}}\times 100\;\mu \text{mol}/\text{L}\times \text{sample dilution factor}$$

### Statistical Analysis

The SPSS19.0 statistical software was used for data analysis. All the values were expressed as means ± Standard Deviation (SD). Independent sample *t* test was used for comparisons between the two groups, and one-way ANOVA was used for multigroup normal distribution data. The significance level was set at alpha=0.05, and there were significant differences between the two sides at P < 0.05.

## Results

### High Insulin and Glucose Concentrations Have Opposite Effects on the Expression of miR-21, and the PTEN/AKT/eNOS and MAPK/ET-1 Pathways in HGECs

**Figure 1:**
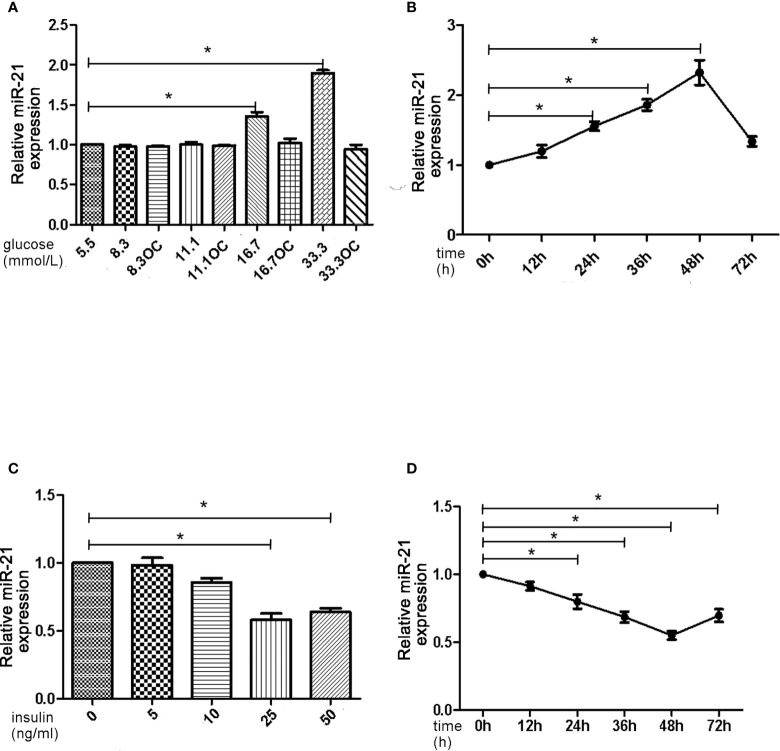
High insulin and glucose concentrations have opposite effects on the expression of miR-21. **(A)**: qRT-PCR analysis of miR-21 in HGECs after treatment with different concentrations of glucose for 48 h. **(B)**: qRT-PCR analysis of miR-21 in HGECs treated with 33.3 mmol/L glucose for different periods of time. **(C)**: qRT-PCR analysis of miR-21 in HGECs after treatment with different concentrations of insulin for 48 h. **(D)**: qRT-PCR analysis of miR-21 in HGECs treated with 50 ng/mL insulin for different periods of time. Data are presented as means ± SD, *P < 0.05. All data are representative of three independent experiments. OC, osmotic control. qRT-PCR, quantitative real-time polymerase chain reaction.

**Figure 2:**
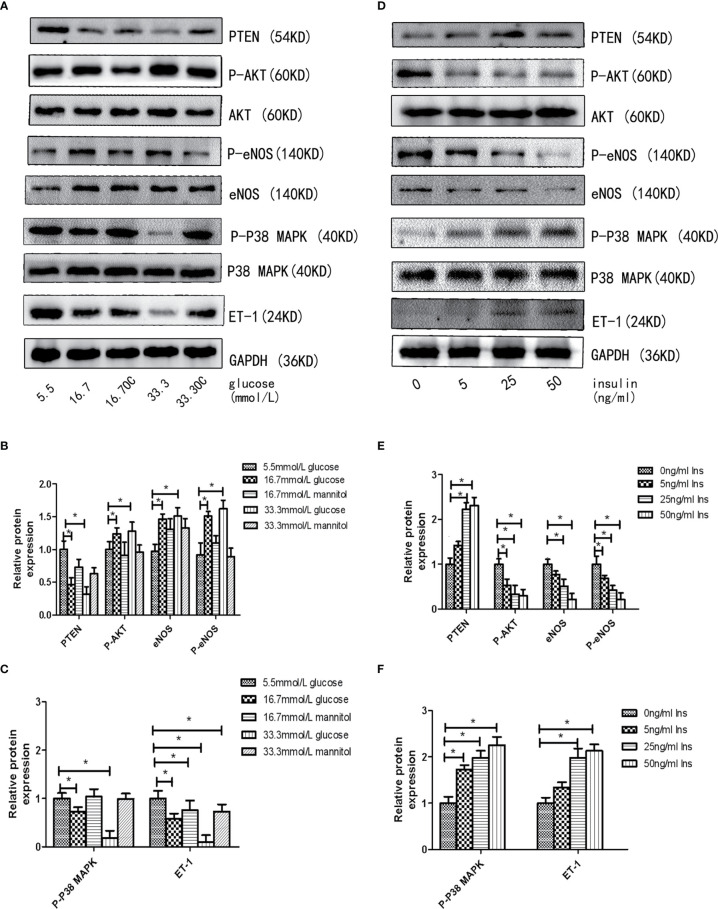
High insulin and glucose concentrations regulate the activity of PTEN/AKT/eNOS MAPK/ET-1 pathways. **(A)**: Westernblot analysis of PTEN, AKT, P-AKT, eNOS, P38 MAPK, P-P38 MAPK and ET-1 expression in HGECs treatment with different concentrations of glucose and 0ng/ml concentration of insulin for 48h. **(B, C)**: Quantification of result in **(A). (D)**: Westernblot analysis of PTEN, AKT, P-AKT, eNOS, P38, P-P38 and ET-1 expression in HGECs treatment with different concentrations of insulin and 5.5mmol/L concentration of glucose for 48h. **(E, F)**: Quantification of result in **(D)**. Data are reported as mean ± SD *P < 0.05. All data are representative of three independent experiments. OC, osmotic control group; Glu, glucose; Ins, insulin; PTEN, phosphatase and tensin homologue on chromosometen; P-AKT, phospho-AKT; P38-MAPK, p38 mitogen-activated protein kinase; P-P38 MAPK, phospho-p38 mitogen-activated protein kinase; eNOS, endothelial nitric oxide synthase; P-eNOS, phospho-endothelial nitric oxide synthase; ET-1, endothelin-1.

First, we assessed the effects of different concentrations of glucose and insulin on the expression of miR-21 in HGECs. The expression of miR-21 increased 1.5-fold in 16.7 mmol/L concentration of glucose and 2-fold in 33.3 mmol/L concentration of glucose. Whereas the expression of miR-21 decreased 0.5-fold in 25ng/ml and 0.4-fold in 50ng/ml concentration of insulin (**Figures 1A–D**). Next, we investigated the effects of high concentrations of glucose and insulin on the expression of proteins in the PTEN/AKT/eNOS and P38-MAPK/ET-1 pathways. High concentration of glucose (>16.7 mmol/L) activated the PTEN/AKT/eNOS pathway and inhibited the P38-MAPK/ET-1 pathway, increasing the expression of P-AKT, eNOS (1.5-fold in 16.7 mmol/L and 1.6-fold in 33.3 mmol/L glucose) and P-eNOS (1.5-fold in 16.7 mmol/L and 1.7-fold in 33.3 mmol/L glucose) and decreasing the expression of PTEN (0.6-fold in 16.7 mmol/L and 0.4-fold in 33.3 mmol/L glucose), P38-MAPK (0.7-fold in 16.7 mmol/L glucose and 0.4-fold in 33.3 mmol/L glucose) and ET-1 (0.6-fold in 16.7 mmol/L glucose and 0.3-fold in 33.3 mmol/L glucose) (**Figures 2A–C**). In contrast, high concentration of insulin (>25 ng/mL) activated the P38-MAPK/ET-1 pathway and inhibited the PTEN/AKT/eNOS pathway, decreasing the expression of P-AKT, eNOS (0.6-fold in 25ng/ml insulin and 0.3-fold in 50ng/ml insulin) and P-eNOS (0.5-fold in 25ng/ml insulin and 0.3-fold in 50ng/ml insulin) and increasing the expression of PTEN(2.4-fold in 25ng/ml insulin and 2.5-fold in 50ng/ml insulin), P38-MAPK (2.1-fold in 25ng/ml insulin and 2.4-fold in 50ng/ml insulin) and ET-1 (2.2-fold in 25ng/ml insulin and 2.3-fold in 50ng/ml insulin) (**Figures 2D–F**).

### Effects of Combined Treatment of HGECs With High Concentrations of Glucose and Insulin on the Expression of MiR-21, PTEN/AKT/eNOS, and MAPK/ET-1 Pathway

**Figure 3:**
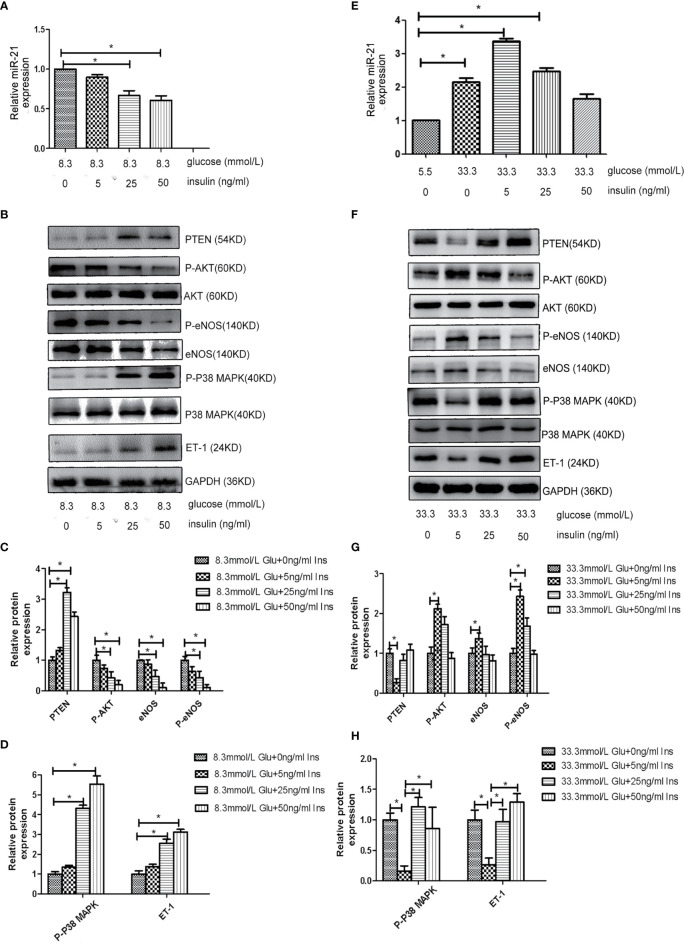
Effects of combined treatment of HGECs with high concentrations of glucose and insulin on the expression of miR-21, PTEN/AKT/eNOS, and MAPK/ET-1 pathways. **(A)**: qRT-PCR analysis of miR-21 in HGECs treated with 8.3 mmol/L of glucose together with different concentrations of insulin. **(B)**: Protein expression of the PTEN/AKT/eNOS and MAPK/ET-1 pathways in groups in **(A). (C, D)**: Quantification of result in **(B). (E)**: qRT-PCR analysis of miR-21 in HGECs treated with 33.3 mmol/L of glucose together with different concentrations of insulin. **(F)**: Protein expression of the PTEN/AKT/eNOS and MAPK/ET-1 pathways in groups in **(E). (G, H)**: Quantification of result in **(F)**. Data are reported as mean ± SD, *P < 0.05. All data are representative of three independent experiments. Glu, glucose; Ins, insulin.

Because both hyperglycemia and hyperinsulinemia exist in patients in the IGT stage, we investigated the change in the expression of miR-21 in HGECs exposed to high concentrations of glucose and insulin in combination. To simulate the physiological environment in the IGT stage *in vitro*, we treated HGECs with 8.3 and 33.3 mmol/L of glucose together with different concentrations of insulin. We observed that the expression of miR-21 was downregulated with the increase in insulin concentration in cells treated with 8.3 mmol/L glucose(decreased 0.7-fold in 8.3 mmol/L glucose +25ng/ml insulin and 0.5-fold in 8.3 mmol/L glucose +50ng/ml insulin group) (**Figure 3A**). The inhibition of PTEN/AKT/eNOS and activation of P38-MAPK/ET-1 pathways enhanced with the increase in insulin concentration (**Figures 3B–D**), the expression of eNOS and P-eNOS decreased (0.7-fold in 8.3 mmol/L glucose +25ng/ml insulin group and 0.3-fold in 8.3 mmol/L glucose +50ng/ml insulin group) and the expression of ET-1 increased (2.8 fold in 8.3 mmol/L glucose +25ng/ml insulin group and 3.3-fold in 8.3 mmol/L glucose +50ng/ml insulin group). In cells treated with 33.3 mmol/L of glucose, the expression of miR-21 in HGECs was upregulated 3.4-fold in 5ng/mL insulin, 2.4-fold in 25ng/mL insulin, and 1.7-fold in 50ng/mL insulin compared to 5.5 mmol/L glucose +0ng/ml insulin group (**Figure 3E**). The activation of PTEN/AKT/eNOS and inhibition of P38-MAPK/ET-1 pathways were found in 33.3 mmol/L glucose +5 ng/ml insulin group (the expression of eNOS and P-eNOS increased 1.4-fold and 2.4-fold separately, the expression of ET-1 decreased 0.3-fold) and 25 ng/mL insulin group (the expression of P-eNOS increased 1.7-fold) compared to 33.3 mmol/L glucose +0ng/ml insulin group, but significant change in the activation of downstream pathways was not found in 33.3 mmol/L glucose +50 ng/mL group insulin compared to 33.3 mmol/L glucose +0ng/ml insulin group. (**Figures 3F–H**).

### The Expression of miR-21 Directly Regulates the Activity of PTEN/AKT/eNOS and MAPK/ET-1 Pathways

**Figure 4:**
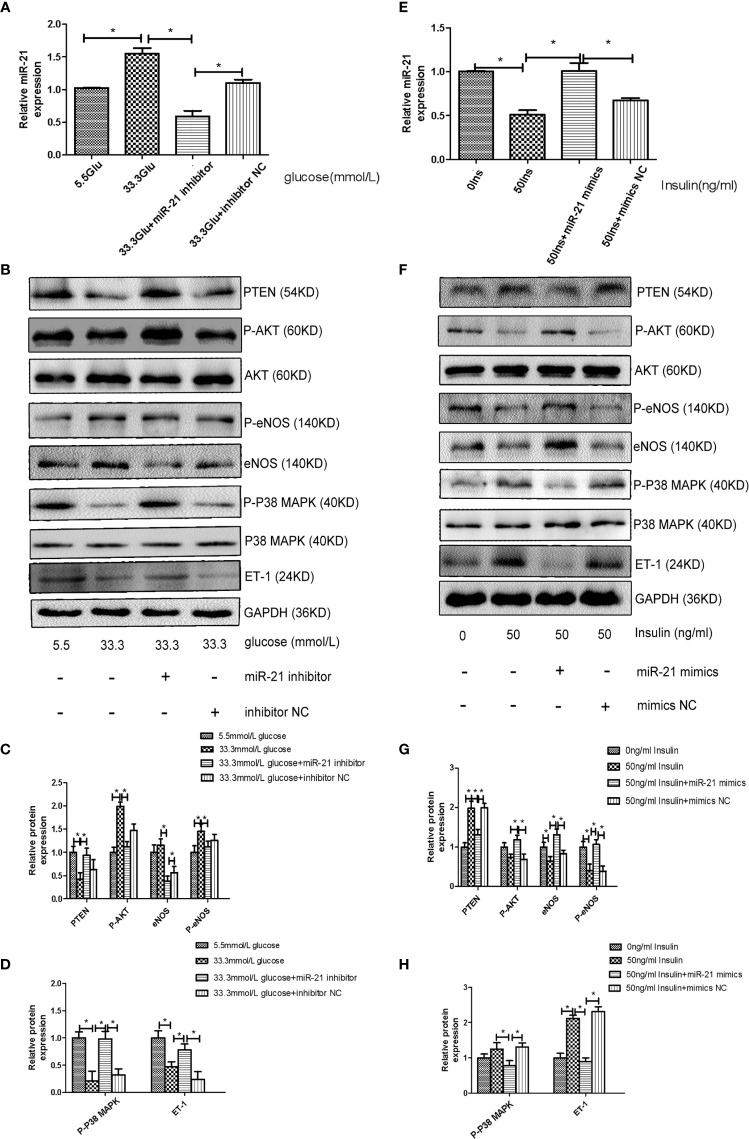
MiR-21 inhibitor/mimics blocked the impact that high concentration glucose/insulin had on downstream pathways. **(A)**: qRT-PCR analysis of miR-21 in HGECs transfected with miR-21 inhibitor or inhibitor NC and treated with different concentrations of glucose. **(B)**: Westernblot analysis of PTEN/AKT/eNOS and MAPK/ET-1 pathways in groups in **(A). (C, D)**: Quantification of result in **(B)**. **(E)**: MiR-21 levels in HGECs transfected with miR-21 mimics or mimics NC and treated with different concentrations of insulin. **(F)**: Westernblot analysis of PTEN/AKT/eNOS and MAPK/ET-1 pathways in groups in **(E)**. **(G, H)**: Quantification of result in **(F)** Data are reported as mean ± SD, *P < 0.05. All data are representative of three independent experiments. Glu, glucose; Ins, insulin; NC, normal control.

**Figure 5:**
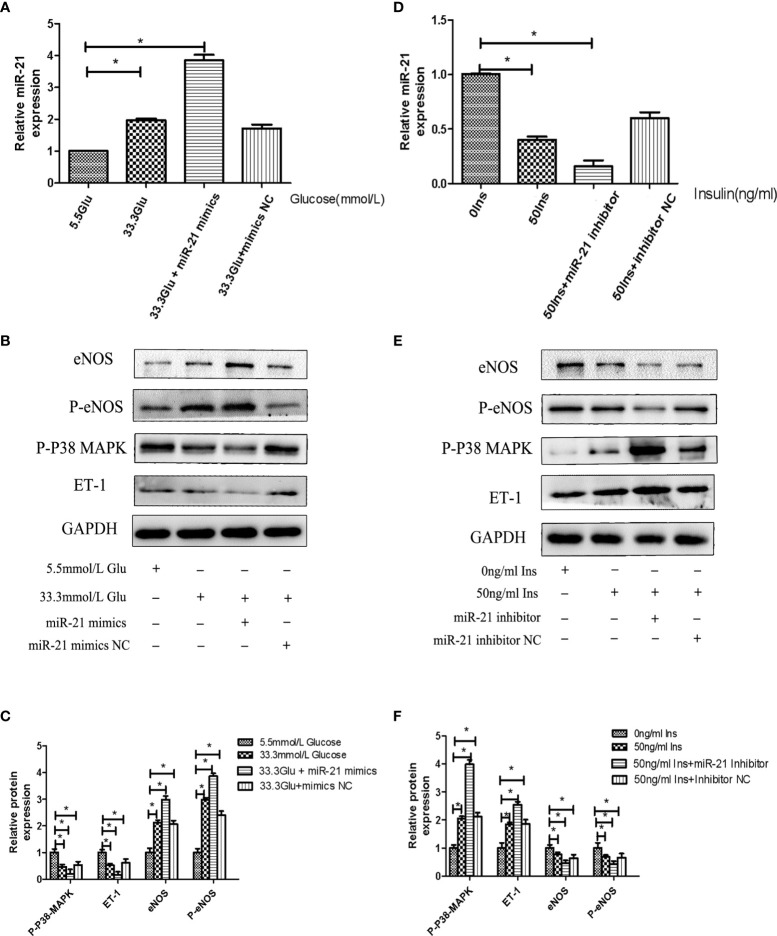
MiR-21 mimics/inhibitor enhanced the impact that high concentration glucose/insulin had on downstream pathways. **(A)**: qRT-PCR analysis of miR-21 in HGECs transfected with miR-21 mimics or mimics NC and treated with different concentrations of glucose. **(B)**: Westernblot analysis of PTEN/AKT/eNOS and MAPK/ET-1 pathways in groups in **(A). (C)**: Quantification of result in **(B). (D)** MiR-21 levels in HGECs transfected with miR-21 inhibitor or inhibitor NC and treated with different concentrations of insulin. **(E)**: Westernblot analysis of PTEN/AKT/eNOS and MAPK/ET-1 pathways in groups in **(D). (F)** Quantification of result in **(E)** Data are reported as mean ± SD, *P < 0.05. All data are representative of three independent experiments. Glu, glucose; Ins, insulin; NC, normal control.

To decipher the role of miR-21 in mediating the regulatory effects of insulin and glucose on the insulin pathway, we used miR-21 inhibitor/mimics for generating cells with loss of function and overexpression of miR-21, respectively. The results of western blot showed that miR-21 inhibitor blocked the activation of the PTEN/AKT/eNOS pathway and the inhibition of the MAPK/ET-1 pathway by glucose (**Figures 4A–D**), but miR-21 mimics enhanced the activation of the PTEN/AKT/eNOS pathway and the inhibition of the MAPK/ET-1 pathway by glucose (**Figures 5A–C**). In contrast, miR-21 mimics blocked the activation of the P38-MAPK/ET-1 pathway and inhibition of the PTEN/AKT/eNOS pathway by insulin (**Figures 4E–H**), but miR-21 inhibitor enhanced the activation of the P38-MAPK/ET-1 pathway and inhibition of the PTEN/AKT/eNOS pathway by insulin (**Figures 5D–F**).

### High Concentrations of Glucose and Insulin Have Opposite Effects on NO and ET-1 Secretion in HGECs

**Figure 6:**
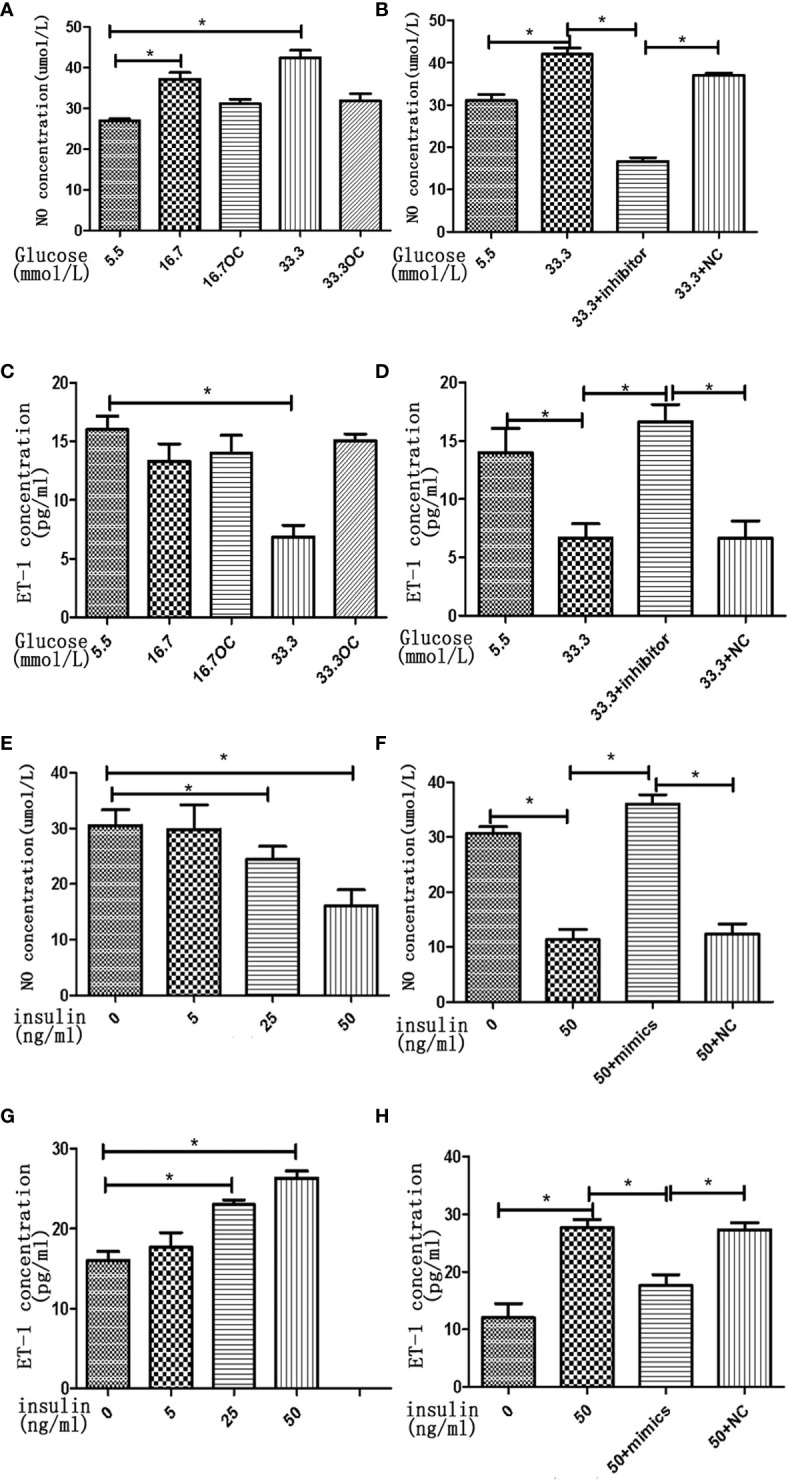
High concentrations of glucose and insulin regulate NO and ET-1 secretion in HGECs. **(A)**: NO seretion of HGECs treated with different concentrations of glucose and 0ng/ml concentration of insulin for 48h. **(B)**: NO seretion of HGECs transfected with miR-21 inhibitor or inhibitor NC and treated with different concentrations of glucose and 0ng/ml concentration of insulin for 48h. **(C)**: ET-1 secretion of HGECs in groups in **(A). (D)**: ET-1 secretion of HGECs in groups in **(B). (E)** NO seretion of HGECs treated with different concentrations of insulin and 8.3mmol/L concentration of glucose for 48h. **(F)**: NO seretion of HGECs transfected with miR-21 mimics or mimics NC and treated with different concentrations of insulin and 8.3mmol/L concentration of glucose for 48h. **(G)**: ET-1 secretion of HGECs in groups in **(E). (H)** ET-1 secretion of HGECs in groups in **(F)**. Data are reported as mean ± SD, *P < 0.05. All data are representative of three independent experiments. OC,osmotic control; NC, normal control; NO, nitric oxide; ET-1, endothelin-1; inhibitor, miR-21 inhibitor; mimics, miR-21 mimics.

To assess the effects of high concentrations of glucose and insulin on the secretory function of endothelial cells, we measured the secretion of NO and ET-1 in HGECs treated with glucose and insulin. High concentration of glucose was found to stimulate the secretion of NO and inhibit the secretion of ET-1 in HGECs in a dose-dependent manner, the secretion of NO increased 1.5-fold in 16.7 mmol/L glucose and 2-fold in 33.3mmol/L glucose and the secretion of ET-1 decreased 0.8-fold in 16.7 mmol/L glucose and 0.5-fold in 33.3mmol/L glucose (**Figures 6A–D**). High concentration of insulin had an opposite effect on the secretion of NO and ET-1, the secretion of NO decreased 0.8-fold in 25ng/ml insulin and 0.5-fold in 50ng/ml insulin and the secretion of ET-1 increased 1.4-fold in 25ng/ml insulin and 1.6-fold in 50ng/ml insulin (**Figures 6E–H**).

## Discussion

The results presented here show that at high concentrations, glucose and insulin have opposite regulatory effects in HGECs, and these effects constitute a dynamic equilibrium. Glucose increased the expression of miR-21 in a concentration-dependent manner, activated the PTEN/AKT/eNOS pathway, and inhibited the MAPK/ET-1 pathway, increased the secretion of NO, and decreased the secretion of ET-1. In contrast, insulin inhibited the PTEN/AKT/eNOS pathway and activated the MAPK/ET-1 pathway, decreased the secretion of NO and increased the secretion of ET-1. Using 8.3 mmol/L glucose and 25 ng/mL insulin to imitate the pathological state of IGT, we found that the effect of high insulin concentration on the endothelial cells was more dominant than that of glucose, resulting in the decrease in the synthesis of NO and increase in the synthesis of ET-1. This might lead to the dysfunction of microcirculatory endothelial cells around renal tubules.

The capillaries around renal tubules are important for renal microcirculation. They are responsible for blood flow and participate in the reabsorption of renal tubules. Observations using electron microscopy have revealed that peritubular capillaries mainly consist of endothelial cells lined by the basement membrane, parietal cells, and a thin stroma (21, 22). In addition to acting as a barrier, endothelial cells are also the source and target of cytokines. These cells can interact with the plasma factors through specific receptors on their membrane, and express vasoactive factors (such as ET-1 and NO) to regulate the blood circulation through peritubular capillaries (23). ET-1 is a potent vasoconstrictor whereas NO is an important vasodilator (24, 25). These two factors ensure a balance between vasoconstriction and vasodilation to regulate the blood flow. The normal functioning of endothelial cells is crucial for maintaining normal microcirculation in the renal tubules. The insulin-signaling pathway within endothelial cells is responsible for the regulation of ET-1 and NO. Upon binding of insulin receptors with insulin, insulin receptor substrate-1 phosphorylates and activates the PI3K/AKT and MAPK pathways, thereby regulating the expression and secretion of ET-1 and NO (26, 27). There are also interactive regulatory networks between the PI3K/AKT and MAPK pathways, which regulate their relative activities and adjust the balance between ET-1 and NO levels (28, 29). Therefore, when endothelial cells are affected by the pathological factors in the IGT stage, they are very likely to dysfunctional in secretion of vasoactive factors, which may lead to a disorder in the renal tubular microcirculation.

Insulin was initially believed to be a vasodilator because of its activation on NO secretion (30, 31). However, in recent studies, insulin-induced vasodilation was confirmed to be impaired in insulin-resistant patients, and patients with obesity or insulin resistance have elevated ET-1 concentrations in the blood (32, 33). The mechanism underlying the direct vascular effect of insulin on endothelial cells remains controversial. In contrast to the findings in previous studies, Ferri C et al. found that insulin stimulates the expression and synthesis of ET-1 and directly enhances the biological effect of ET-1 in endothelial cells *in vitro* (34). In another study, it was shown that insulin can activate the expression of both NO and ET-1 (35). Moreover, a study on rat mesenteric vessels showed that the ratio of the expression of NO and ET-1 changes with the dose of insulin (36). To unravel the vascular effect of insulin, we used different concentrations of insulin to interfere with the glomerular endothelial cells. We observed that a high concentration (>25 ng/mL) of insulin independently stimulated the synthesis and secretion of ET-1, attenuated the synthesis of eNOS and secretion of NO, and that this effect was concentration- and time-dependent (within 48 h). We believe that there are two main reasons for the above controversy: 1. The effect of insulin on NO secretion is determined by insulin concentration and incubation time. Derek A, et al. has confirmed through experiments that the increase in NO secretion caused by insulin was due to short-term ETB receptor activation, but the increase in NO secretion caused by ETB receptor activation will be weakened by the increase of insulin concentration and incubation time (10min), eventually, lead to a decrease in NO secretion and an increase in ET-1 secretion (37). In our experiments, the endothelial cells in the high-concentration insulin group were cultured in high insulin concentrations (>25ng/ml) for a long time (48 hours), resulting in the decrease of NO secretion. 2. The sources and types of endothelial cells used in these studies are different. In most literature, aortic endothelial cells and umbilical vein endothelial cells were used for experiments, while in our experiments we use glomerular endothelial cells. Endothelial cells of different sources and types are different in morphology, structure, and function (38). Therefore, when stimulated by insulin, the secretion function of glomerular endothelial cells may be different from other types of cells. Our results suggest that insulin exerts independent vasoconstriction effects by regulating the secretory function of glomerular endothelial cells, and that the vasoconstriction effect is concentration- and time- dependent.

The damage of endothelial cells caused by high glucose concentrations has been widely studied. High concentration of glucose can lead to the activation of PKC and COX-2, resulting in the overproduction of angiotensin (AngII), overexpression of TGF-β, endothelial cell apoptosis, abnormal NO activity, and increase in the generation of ROS, which increases the permeability of glomerular filtration barrier, leading to glomerular hyperfiltration (39–43). At the same time, the glomerulus would expand itself to adapt to the hyperfiltration while pulling the mesangial cells, so that the mesangial cells increase collagen IV, V, I, II, fibronectin and laminin synthesis. A large amount of ECM accumulates causing progressive damage to the glomerulus, which eventually develops into irreversible glomerulosclerosis (44). However, the studies cited above focused on the effect of high concentration of glucose (>25 mmol/L in most of the studies) on endothelial cells. It is still not clear whether a mild increase in the concentration of glucose in the IGT stage has the same effect. In this study, we observed the effect of different concentrations of glucose on endothelial cells. There was no significant change in the expression of ET-1 and NO proteins in glomerular endothelial cells treated with glucose at concentrations lower than 11.1 mmol/L. However, the synthesis of eNOS and secretion of NO were upregulated, and the synthesis and secretion of ET-1 were downregulated in glomerular endothelial cells treated with glucose at concentrations higher than 16.7 mmol/L, suggesting that the effect of glucose on endothelial cells is concentration dependent. At glucose concentrations lower than 16.7 mmol/L, the function of endothelial cells was not significantly affected.

**Figure 7:**
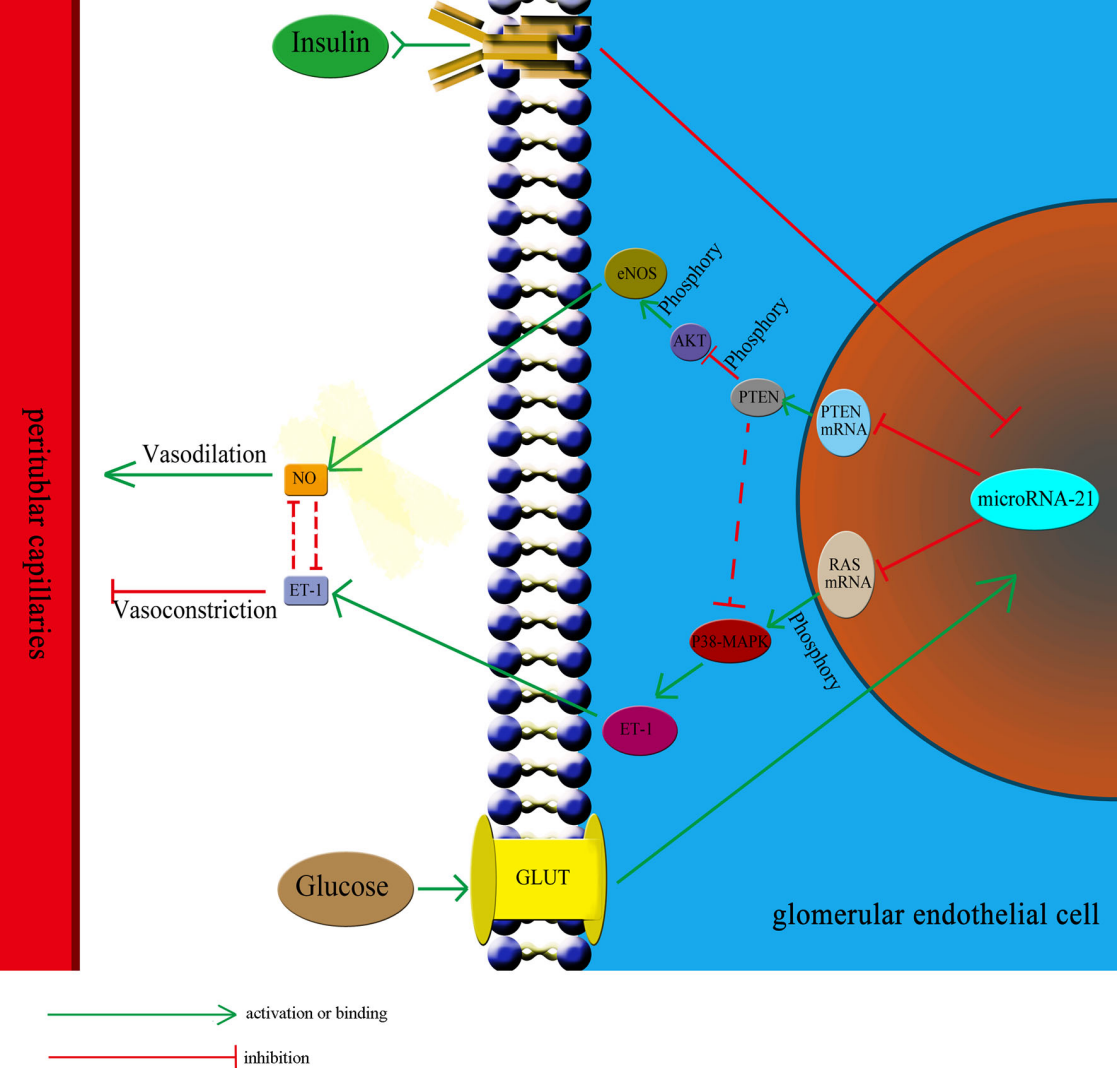
The relative balance of of insulin and glucose regulates the function of HGECs and peritubular capillaries. The relative balance of insulin and glucose regulates the balance of the PTEN/AKT/eNOS and P38-MAPK/ET-1 pathways protein expression by affecting the expression of miR-21, which in turn affects the balance between the secretion of ET-1 and NO and regulate the blood flow through peritubular capillaries. The unbalance of insulin and glucose would have cascading effect on the expression of downstream proteins and lead to unbalanced secretion of ET-1 and NO, resulting in ischemia and hypoxia in renal tubules. GLUT, glucose transporter.

IGT represents an intermediate stage in the progression from NGT to diabetes, in which plasma glucose levels are above normal but do not meet the criteria for diabetes (45, 46). According to the diagnostic criteria set by the World Health Organization, IGT is defined by fasting plasma glucose concentrations lower than 7 mmol/L and OGTT plasma glucose concentrations higher than 7.8 mmol/L but lower than 11.1 mmol/L. To respond to elevated plasma glucose concentration and insulin resistance, β-cells secrete large amounts of insulin, which results in hyperinsulinemia (44). Therefore, hyperinsulinemia and hyperglycemia are two independent pathological factors existing in the IGT stage that could affect the functioning of glomerular endothelial cells. In this study, we observed the effect of insulin and glucose on the function of endothelial cells to be opposite. Thus, it is difficult to predict the effect of the two in combination. To investigate this, we treated HGECs with glucose at three different concentrations: 5.5 mmol/L (representing the normal glucose concentration), 8.3 mmol/L (slightly increased glucose concentration, representing the IGT stage), and 33.3 mmol/L (representing significantly increased glucose concentration). Subsequently, we examined the expression of miR-21 when these cells were cotreated with different concentrations of insulin (0 ng/mL, representing no insulin; 5 ng/mL, representing normal insulin concentration; 25 and 50 ng/mL, representing for high insulin concentrations). The results showed that in cells treated with 5.5 and 8.3 mmol/L glucose, 25 or 50 ng/mL of insulin strongly decreased the expression of miR-21 compared with 0 and 5 ng/mL insulin. We speculate that this was because, at 5.5 and 8.3 mmol/L concentrations, glucose had a less stimulatory effect on the expression of miR-21, but 25 or 50 ng/mL of insulin strongly inhibited the expression of miR-21. Thus, the stimulatory effect of glucose is much weaker than the inhibitory effect of insulin, leading to an inhibition in the expression of miR-21. At a concentration of 33.3 mmol/L, the stimulatory effect of glucose was enhanced compared with 8.3 mmol/L of glucose, thereby alleviating the inhibitory effect of insulin, increasing the expression of miR-21 in cells treated with 25 and 50 ng/mL insulin. Hence, the combined effect of glucose and insulin on endothelial cells depends on their relative concentrations. Overall, we can conclude that the effect of insulin and glucose on endothelial cells depends on a multilevel, dynamic balance involving gene expression, protein synthesis, and cytokine secretion. The relative balance of insulin and glucose regulates the balance of the PI3K/AKT/eNOS and MAPK/ET-1 pathways and protein expression by affecting the expression of miR-21, which in turn affects the balance between the secretion of ET-1 and NO. In this complex multilevel regulation, once the balance between insulin and glucose is broken, there is a cascading effect on the balance of expression of downstream proteins and secretion of cytokines, which ultimately leads to the unbalanced secretion of ET-1 and NO, resulting in ischemia and hypoxia in renal tubules (**Figure 7**).

Our experiment also simulated the pathophysiological elements in different periods *in vitro*: in the IGT stage, blood glucose concentration did not increase significantly (8.3mmol/L), but insulin concentration increased significantly (as shown in **Figure 3A**, 8.3mmol/L glucose + 25ng/ml insulin group and 8.3 mmol/L glucose+50ng/ml insulin group), glucose had little impact on the expression of miR-21, while high concentration insulin had an obvious inhibitory effect on the expression of miR-21. In diabetes, blood glucose significantly increased, but the function of islets was impaired, and insulin secretion was decreased (as shown in **Figure 3G**, the 33.3mmol/L glucose + 0ng/ml insulin and 33.3mmol/L glucose + 5ng/ml insulin groups), at this time, the effect of insulin on the expression of miR-21 was weakened, but high concentration of glucose up-regulated the expression of miR-21 significantly. Through the above results, we can conclude that hyperinsulinemia is the main pathological factor leading to abnormal expression of miR-21 in endothelial cells in the IGT stage. Therefore, it is necessary to take measures to reduce the blood insulin concentration in the IGT stage to prevent IGT nephropathy.

Besides, in the 33.3 mmol/L glucose group, we found that normal concentration of insulin (0-5ng/ml) can further enhance the expression of miR-21, while a higher (> 25ng/ml) concentration of insulin can inhibit the expression of miR-21. In the existing literature, it has been confirmed that normal concentration of insulin can improve the uptake of glucose by promoting the translocation of GLUT4, but in the insulin resistance animal models, the promoting effect of insulin on GLUT4 disappeared (47). Some literature suggested that this was related to the decreased synthesis of insulin receptors and the abnormal insulin binding (48). So the cooperative mode of insulin and glucose depends on the concentration of insulin. A normal concentration of insulin can enhance the effect of high concentration of glucose by promoting glucose uptake, while a higher concentration of insulin (>25ng/ml) in the IGT stage counteracts the effects of glucose by inhibiting miR-21 expression.

MicroRNAs are non-coding single-stranded RNA molecules encoded by endogenous genes. Mature microRNAs can have complementary base pairing with the 3′-noncoding regions of target genes, and inhibit the translation of target proteins (49, 50). MiR-21 is an important miRNA located on chromosome 17q23.2, which was initially found to be highly expressed in cancers (47). Subsequent research has shown that miR-21 also participates in the pathological progress of diabetic nephropathy, such as in interstitial fibrosis, endothelial cell apoptosis, and overexpression of inflammatory factors (51–54). In this study, we found that in glomerular endothelial cells, the expression of miR-21 was increased in the presence of a high concentration of glucose and was decreased when the cells were treated with insulin at a high concentration. The expression of miR-21 determines the activity of the downstream insulin pathways and the expression of cytokines, indicating that it is the key regulator in mediating the effects of glucose and insulin on downstream proteins and the secretory functions of endothelial cells. Under conditions of hyperglycemia and hyperinsulinemia, the expression of miR-21 in glomerular endothelial cells depends on the relative concentrations of glucose and insulin.

Our results also indicate that the change in the expression of miR-21 may reflect the early dysfunction of endothelial cells, and could serve as a target molecule for early diagnosis and treatment of IGT nephropathy. In recent studies, researchers took blood and urine samples of diabetes patients and determined the amount of NO by measuring the ratio of nitrite/nitrate (55) or used ELISA kits to determine the amount of ET-1 secretion (56), but the above methods to assess endothelial function are very complicated and the results are unstable. Some new techniques, such as brachial artery flow-meditated dilatation (FMD), have been developed to assess the function of endothelial cells, but their value is still debated (57). In our study, we can see that the expression of miR-21 can reflect the balance between NO and ET-1: the more mir-21 expressed, the more NO and less ET-1 is secreted. Therefore, we speculate that the expression of miR-21 in urine can reflect the function of renal endothelial cells. Besides, in our previous study, we have already performed Agilent miRNA microarray analysis to detect some miRNA expression in urine in IGT patients, the result showed some particular miRNA had significantly up-regulated (58). In subsequent experiments, we will collect urine samples from IGT patients and try to verify the above hypothesis.

In conclusion, changes in glucose and insulin concentrations in the IGT stage affect the secretory function of renal endothelial cells, which impairs the regulation of blood flow through peritubular capillaries, and finally leads to ischemia and hypoxia in renal tubules. To prevent DKD, it is necessary to prevent the dysfunctioning of renal endothelial cells in the IGT stage. Besides, the expression of miR-21 changes significantly in the IGT stage and is involved in the pathological mechanism of IGT nephropathy. Therefore, miR-21 could be a sensitive index to predict renal damage and could be a potential target for the prevention of renal damage in the IGT stage.

## Data Availability Statement

The raw data supporting the conclusions of this article will be made available by the authors, without undue reservation.

## Author Contributions

RL performed the research, analyzed data and wrote the manuscript. BC analyzed data. ZGG, SG, JW, JX, ZH, YZ, SY, ZHG, JY, HS, and BC designed research and revised the manuscript. All authors contributed to the article and approved the submitted version.

## Funding

The author(s) disclosed receipt of the following financial support for the research, authorship, and/or publication of this article: This work was supported by the National Key R&D Program of China (2018YFC1314000), National Natural Science Foundation of China (81774043, 82074253), Tianjin Science and Tecnology Program (17ZXMFSY00140).

## Conflict of Interest

The authors declare that the research was conducted in the absence of any commercial or financial relationships that could be construed as a potential conflict of interest.
