# Supplementary material for: Pathological Hyperinsulinemia and Hyperglycemia in the Impaired Glucose Tolerance Stage Mediate Endothelial Dysfunction Through miR-21, PTEN/AKT/eNOS, and MARK/ET-1 Pathways
Source: Front Endocrinol (Lausanne). 2021 Apr 23;12:644159. doi: 10.3389/fendo.2021.644159 (PMC8104127; doi:10.3389/fendo.2021.644159)
Supplement: Supplementary file 1 [file DataSheet_1.pdf]

Sub figure1 the Ct value of U6 in each group

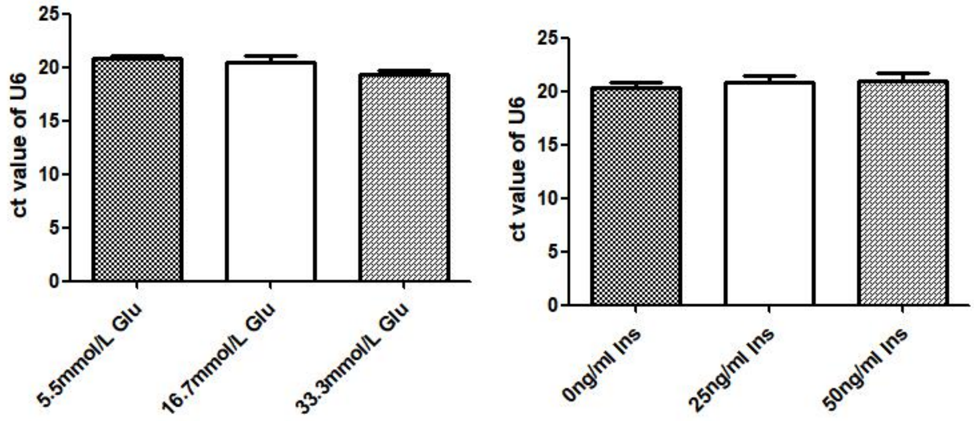

Sub figure2 the phosphorylation of eNOS in glomerular endothelial cells treated with different concentrations of glucose or mannitol

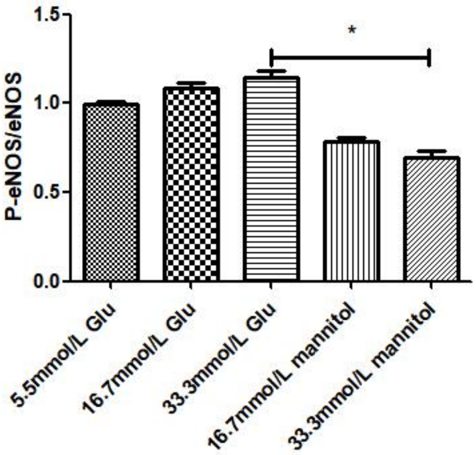

Sub figure3 the expression of miR-21 in endothelial cells transfected by miR-21

mimics in 5.5mmol/l glucose +0ng/ml insulin

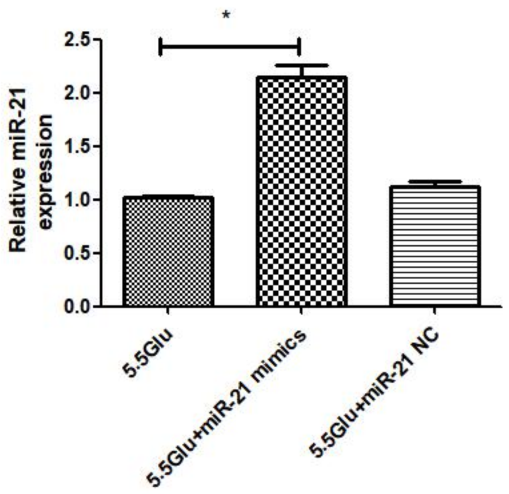

Sub figure4 scratch assay result of HECGs treated with high concentration of glucose or insulin

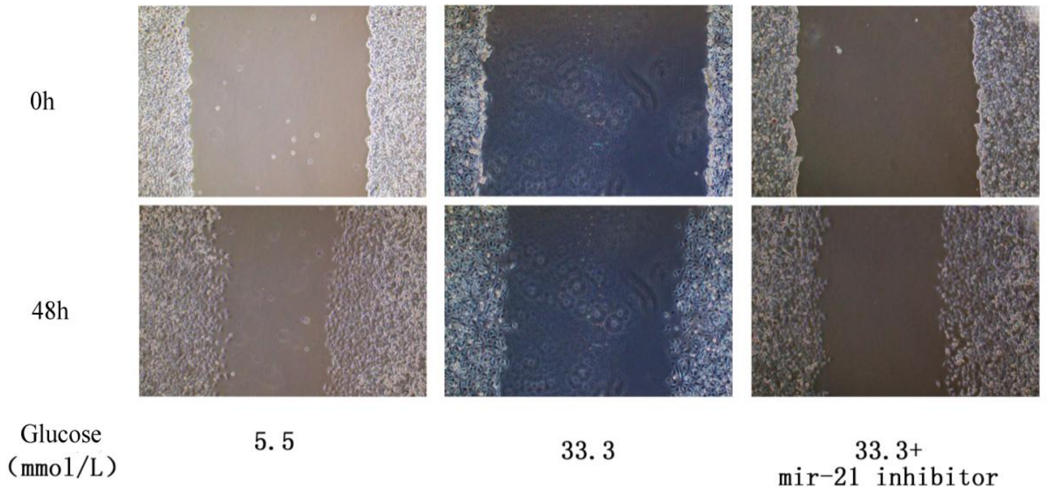

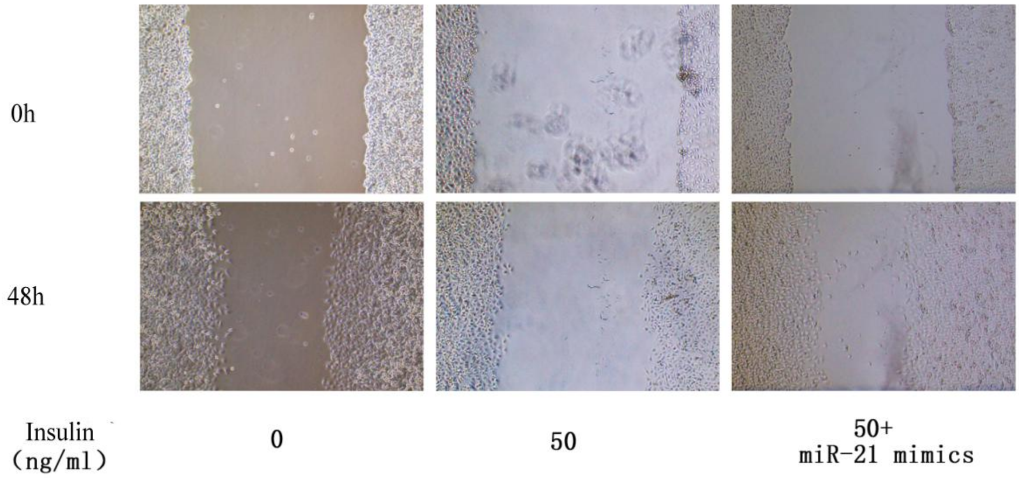
0h
48h
Insulin
（ng/ml）
0
50
50+
miR-21 mimics
